# Empirical comparison and analysis of machine learning-based approaches for druggable protein identification

**DOI:** 10.17179/excli2023-6410

**Published:** 2023-08-29

**Authors:** Watshara Shoombuatong, Nalini Schaduangrat, Jaru Nikom

**Affiliations:** 1 Center for Research Innovation and Biomedical Informatics, Faculty of Medical Technology, Mahidol University, Bangkok, Thailand, 10700; 2 Research Methodology and Data Analytics Program, Faculty of Science & Technology, Prince of Songkla University, Pattani, Thailand, 94000

**Keywords:** druggable proteins, sequence analysis, bioinformatics, machine learning, deep learning, ensemble learning

## Abstract

Efficiently and precisely identifying drug targets is crucial for developing and discovering potential medications. While conventional experimental approaches can accurately pinpoint these targets, they suffer from time constraints and are not easily adaptable to high-throughput processes. On the other hand, computational approaches, particularly those utilizing machine learning (ML), offer an efficient means to accelerate the prediction of druggable proteins based solely on their primary sequences. Recently, several state-of-the-art computational methods have been developed for predicting and analyzing druggable proteins. These computational methods showed high diversity in terms of benchmark datasets, feature extraction schemes, ML algorithms, evaluation strategies and webserver/software usability. Thus, our objective is to reexamine these computational approaches and conduct a comprehensive assessment of their strengths and weaknesses across multiple aspects. In this study, we deliver the first comprehensive survey regarding the state-of-the-art computational approaches for *in silico* prediction of druggable proteins. First, we provided information regarding the existing benchmark datasets and the types of ML methods employed. Second, we investigated the effectiveness of these computational methods in druggable protein identification for each benchmark dataset. Third, we summarized the important features used in this field and the existing webserver/software. Finally, we addressed the present constraints of the existing methods and offer valuable guidance to the scientific community in designing and developing novel prediction models. We anticipate that this comprehensive review will provide crucial information for the development of more accurate and efficient druggable protein predictors.

## Introduction

Druggable proteins belong to large protein families identified as suitable drug targets. These proteins exhibit the ability to bind with high affinity to small drug-like molecules, leading to desirable therapeutic effects (Liu and Altman, 2014[25]; Owens, 2007[32]). Approximately 60 % of projects in the drug discovery domain lead to failure due to the target being considered undruggable (Sakharkar et al., 2007[35]). Therefore, the advancement in a drug discovery project, where the precise identification of drug targets is essential, depends on the druggability of a protein (Overington et al., 2006[31]). Analyzing the three-dimensional structure of a protein through experimental methods leads to a lengthy development cycle (Sakharkar et al., 2007[35]). Although traditional experimental approaches are capable of accurately identifying drug targets, they are labor-intensive and not easily adaptable for high-throughput applications. Computational approaches that rely solely on the primary sequences of proteins can serve as a valuable supplement to experimental methods, enabling swift characterization and prediction of druggable proteins. The continuous discovery of novel proteins through next-generation sequencing opens up vast opportunities to identify potential druggable candidates that remain unexplored. Therefore, the accurate and rapid identification of druggable proteins from an extensive pool of sequenced proteins is of utmost importance in the quest for developing new drugs (Lindsay, 2005[23]). 

**Figure 1:**
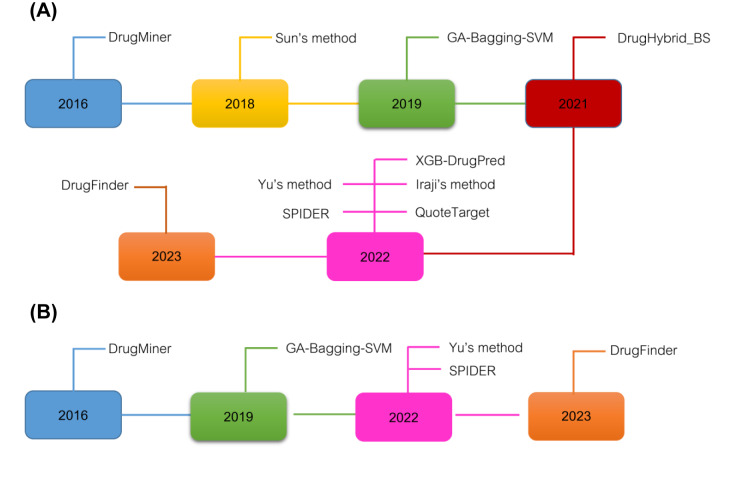
Timeline of the existing state-of-the-art predictors (A) and webserver/software availability (B)

Over the last few decades, numerous attempts have been made to develop data-driven machine learning (ML)-based computational approaches to further the identification and characterization of a variety of potential proteins and peptides in tandem with the experimental techniques (Charoenkwan et al., 2023[5][8]; Hasan et al., 2021[14]; Qiang et al., 2020[33]; Rao et al., 2018[34]; Wang et al., 2019[41]; Wei et al., 2018[44]; Xie et al., 2021[46]). In this field, there are ten existing state-of-the-art computational approaches, including DrugMiner (Jamali et al., 2016[17]), Sun's method (Sun et al., 2018[38]), GA-Bagging-SVM (Lin et al., 2019[22]), DrugHybrid_BS (Gong et al., 2021[13]), XGB-DrugPred (Sikander et al., 2022[36]), Iraji's method (Iraji et al., 2022[16]), Yu's method (Yu et al., 2022[47]), SPIDER (Charoenkwan et al., 2022[10]), QuoteTarget (Chen et al., 2023[12]), and DrugFinder (Zhang et al., 2023[48]). Table 1(Tab. 1) (References in Table 1: Charoenkwan et al., 2022[10]; Chen et al., 2023[12]; Gong et al., 2021[13]; Iraji et al., 2022[16]; Jamali et al., 2016[17]; Lin et al., 2019[22]; Sikander et al., 2022[36]; Sun et al., 2018[38]; Yu et al., 2022[47]; Zhang et al., 2023[48]) provides the information of these ten existing predictors in terms of benchmark datasets, feature extraction schemes, ML strategies, evaluation methods, and webserver availability. Furthermore, the timelines of the existing computational approaches and webserver/software availability are summarized in Figure 1(Fig. 1). 

In this article, we deliver the first comprehensive survey regarding the existing state-of-the-art predictors. Specifically, we cover a variety of multiple important aspects, including benchmark datasets along with feature extraction schemes, ML strategies, evaluation methods, and webserver availability. First, we summarized all benchmark datasets and the three types of ML methods used for the construction and evaluation of the existing state-of-the-art approaches. Second, we investigated the effectiveness of these computational approaches for each benchmark dataset, considering both cross-validation and independent tests. Third, we provided a summary regarding the important features used in this field and the availability of existing webserver/software. Finally, we discussed the current limitations of the existing methods and provided useful guidance to researchers who are interested in developing a more accurate and robust approach in future studies.

## Materials and Methods

### Overall framework of druggable protein identification using machine learning methods

**Figure 2:**
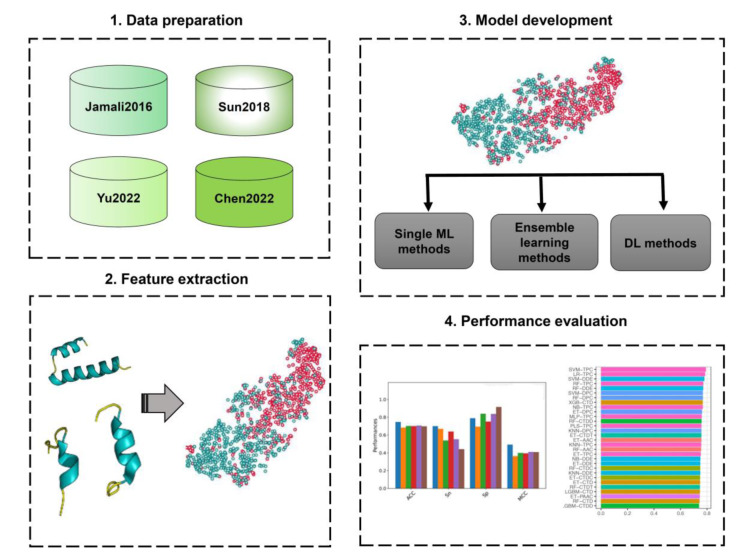
The general machine learning framework of the prediction of druggable proteins

The ML framework of druggable protein identification is summarized in Figure 2(Fig. 2). As can be seen, there are five main stages (Charoenkwan et al., 2021[3], 2022[7]; Hongjaisee et al., 2019[15]). The first stage is to prepare the benchmark training and independent test datasets. The training datasets are used for model training and optimization, while the independent test datasets are used for validating the generalizability and reliability of the models. The second stage is to represent protein sequences into fix-length feature vectors (Qiang et al., 2020[33]; Wei et al., 2018[44]). The third stage involves training and optimization of the prediction model based on several ML frameworks. In the fourth stage, the trained prediction models are evaluated using well-known performance evaluation strategies, such as k-fold cross-validation and independent tests (Arif et al., 2020[1]; Manavalan et al., 2018[29]). Finally, the selected prediction models are implemented as an online webserver.

### Construction of training and independent test datasets

Until now, there are four benchmark datasets that have been used for developing the ten existing state-of-the-art computational approaches, including Jamali2016 (Jamali et al., 2016[17]), Sun2018 (Sun et al., 2018[38]), Yu2022 (Yu et al., 2022[47]), and Chen2022 (Chen et al., 2023[12]). Table 2(Tab. 2) (References in Table 2: Chen et al., 2023[12]; Jamali et al., 2016[17]; Sun et al., 2018[38]; Yu et al., 2022[47]) provides details of these datasets. The Jamali2016 dataset was established by Jamali et al. (2016[17]). This dataset consisted of 1,224 positives and 1,319 negatives. In the Jamali2016 dataset, the positive samples were derived from proteins that are able to interact with drugs, while the negative samples were derived from proteins that cannot be deemed as drug targets. The Jamali2016 dataset was selected to develop six druggable protein predictors (i.e., DrugMiner (Jamali et al., 2016[17]), GA-Bagging-SVM (Lin et al., 2019[22]), DrugHybrid_BS (Gong et al., 2021[13]), XGB-DrugPred (Sikander et al., 2022[36]), Iraji's method (Iraji et al., 2022[16]), and DrugFinder (Zhang et al., 2023[48])). For the Sun2018 dataset, it was introduced by Sun et al. (2018[38]) and comprises two main sub-datasets, including small and large datasets. The positive samples for the small dataset was directly obtained from the Jamali2016 dataset (1,224 positives), while the positive samples for the large dataset was obtained from experimental small molecules' targets based on DrugBank (5,503 positives). The negative samples for the small and large datasets consisted of 1,235 and 5,498 samples, respectively, derived from Swiss-Prot (Boeckmann et al., 2003[2]). Regarding the dataset from Yu2022, it was proposed by Yu et al. (2022[47]) by considering the Jamali2016 dataset as the training dataset, while Yu et al. utilized the DrugBank 5.0 database (Wishart et al., 2018[45]) along with the Kim's study (Kim et al., 2017[18]) to create the independent test dataset containing 224 positives and 237 negatives. The Yu2022 dataset was employed to develop a few druggable protein predictors (i.e., Yu's method (Yu et al., 2022[47]) and SPIDER (Charoenkwan et al., 2022[10])). As for the last benchmark dataset in this field, it was collected from the DrugBank 5.0 database (Wishart et al., 2018[45]) and the Therapeutic Target Database (TTD) (Wang et al., 2020[42]). The Blast tool was used to exclude redundant samples, with E-values of 0.001, 1, and 10 (positives, negatives) resulting in databases of (11,803, 7900), (9,389, 5941), and (5330, 3078), respectively.

### State-of-the-art computational approaches for druggable protein identification

Based on the types of ML methods employed, the existing computational approaches listed in Table 1(Tab. 1) can be categorized into three groups. The first group is developed based on single ML methods, such as neural network (NN), random forest (RF), and eXtreme gradient boosting (XGB). The second group is developed based on ensemble learning methods, such as bagging and stacking strategies; and the third group is developed based on deep learning (DL) methods, such as convolutional neural network (CNN) and recurrent neural network (RNN).

As can be noticed in Table 1(Tab. 1), there are four out of ten existing computational approaches designed using single ML methods, including DrugMiner (Jamali et al., 2016[17]), Sun's method (Sun et al., 2018[38]), XGB-DrugPred (Sikander et al., 2022[36]), and DrugFinder (Zhang et al., 2023[48]). In 2016, DrugMiner was introduced by Jamali et al. (2016[17]) and considered the first sequence-based predictor designed for discriminating druggable proteins from non-druggable proteins. In this method, three feature descriptors, consisting of amino acid composition (AAC), dipeptide composition (DPC), and physicochemical properties (PCP), were used to represent druggable proteins as fix-length feature vectors. Then, Jamali et al. combined these three feature descriptors and represented each sequence with 443-D feature vectors. The Relief method was then used to identify *m* out of 443 features. The high accuracy (ACC) of 0.921 was achieved by using NN in conjunction with the top-130 informative features. For XGB-DrugPred, it was developed based on three well-known feature descriptors (i.e., grouped dipeptide composition (GDPC), reduced amino acid alphabet (RAAA), and pseudo amino acid segmentation (S-PseAAC)). Then, each feature descriptor was optimized using the combination of RFE and XGB. After performing the feature optimization, top-73, top-17, and top-36 information features from RAAA, GDPC, and S-PseAAC, respectively, were determined and integrated to generate the final feature vector. These fnal feature vectors were trained and tested for the performance of ET, RF, and XGB. The high ACC of 0.949 was achieved by using XGB. In case of DrugFinder, it was developed by Zhang et al. (2023[48]). Zhang et al. performed experiments with many ML methods (i.e., XGB, RF, support vector machine (SVM), naive Bayes (NB), and k-nearest neighbors (KNN)) and feature encoding schemes (i.e., Seq2Vec, Prot_T5_Xl_Uniref50 (T5), position-specific scoring matrix (PSSM), and Prot_Bert_BFD). Among the four feature encoding schemes, the T5 model was then selected to perform the feature optimization process. The optimal model of Zhang's study achieving a cross-validation ACC of 0.950, was obtained from the combination of XGB and the top-1500 information features.

The limitation of single ML methods is that their performance was not satisfactory enough for practical applications. Therefore, the goal of ensemble learning methods is to integrate heterogenous weak ML models to create a single hybrid model with a more comprehensive performance. As shown in Table 1(Tab. 1), there are three computational approaches employed the ensemble learning methods to construct the prediction models, including GA-Bagging-SVM (Lin et al., 2019[22]), DrugHybrid_BS (Gong et al., 2021[13]), and SPIDER (Charoenkwan et al., 2022[10]). Specifically, GA-Bagging-SVM and DrugHybrid_BS were developed based on the bagging strategy, while only SPIDER was developed based on the stacking strategy. For the bagging strategy, there are three main steps for the construction of GA-Bagging-SVM and DrugHybrid_BS, including feature representation, feature importance selection, and final model construction. Taking GA-Bagging-SVM as an example, first, three feature descriptors (i.e., PAAC, DPC, and reduced sequence (RS)) were used to represent druggable proteins. The PAAC, DPC, and RS descriptors were defined as 23-D, 400-D, and 163-D feature vectors, respectively. Second, the genetic algorithm (GA) was employed to optimize the original feature vector. Finally, multiple SVM classifiers were integrated to develop a hybrid model using the bagging algorithm. The highest ACC and Matthew's correlation coefficient (MCC) of 0.934 and 0.871 were attained by using top-143 informative features. In case of the stacked model SPIDER, it is known as a stacked ensemble learning model. Specifically, SPIDER involves two main levels of learning processes, where the classifiers developed based on the first and second learning processes are called as the base-classifier and meta-classifier, respectively. For the first step, 60 base-classifiers were created by using six different ML methods, each in conjunction with ten feature encodings. In the second step, all the base-classifiers were employed to generate 60 probabilistic features. These features were represented as a 60-dimensional (60-D) feature vector and used for the construction of the stacked model.

To date, DL method has been known as a cutting-edge technique that is successfully utilized in the field of bioinformatics and computational biology (Charoenkwan et al., 2021[6]; Rao et al., 2018[34]; Wang et al., 2019[41]; Xie et al., 2021[46]). In this field, Table 1(Tab. 1) shows that there are three computational approaches that employed DL methods to construct the prediction models, including Iraji's method (Iraji et al., 2022[16]), Yu's method (Yu et al., 2022[47]), and QuoteTarget (Chen et al., 2023[12]). Among these three druggable protein predictors, Iraji's method is the first druggable protein predictor applied using the DL method (Iraji et al., 2022[16]). In Iraji's method, Iraji et al. created two prediction models using PCPs. In the first prediction model, each protein sequence is encoded into fix-length feature vectors based on the autocovariance method. The six PCPs, including polarity, hydrophilicity, hydrophobicity, polarizability, net charge index of side chain, and solvent-accessible surface area, were applied in this step. As a result, each protein sequence is represented with a 180-D feature vector. The deep stacked sparse auto-encoders (DSSAEs) network determines important features from the 180 features. Then, a set of the important features is translated into a 30-D feature vector. In the second prediction model, the deep CNN was fed the output of DSSAEs.

### Performance evaluation measures

To date, k-fold cross-validation and independent tests have been widely used for the performance evaluation of the existing druggable protein predictors. In the case of the 10-fold cross-validation test, the dataset is divided into 10 sub-datasets. For the 1^st^ iteration, one of the 10 sub-datasets is treated as the 1^st^ testing dataset, while the remaining nine sub-datasets are employed to train the 1^st^ prediction model. Thus, the prediction results of the 1^st^ prediction model will be evaluated based on the 1^st^ testing dataset. As a result, the process of the 10-fold cross-validation test is repeated 10 times. The final performance is obtained from the average performance over 10 individual prediction results. To assess the predictive ability of the existing druggable protein predictors, seven commonly used performance metrics were employed. These include ACC, F1, MCC, sensitivity (Sn), specificity (Sp), area under the receiver operating curve (AUC), and precision (PRE) (Charoenkwan et al., 2022[4][9]; Mandrekar, 2010[30]; Ullah et al., 2021[39]). They are defined as follows:


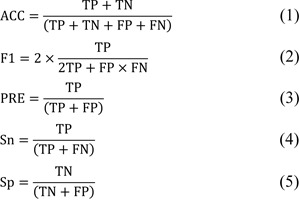


Specifically, TP and TN represent the numbers of true positives and true negatives, respectively, while FP and FN the numbers of false positives and false negatives, respectively (Lai et al., 2019[19]; Lv et al., 2020[28], 2021[27]; Su et al., 2018[37]).

## Results and Discussion

### Comparative assessment and analysis

Among the four benchmark datasets, Jamali2016 (Jamali et al., 2016[17]), Yu2022 (Yu et al., 2022[47]), and Chen2022 (Chen et al., 2023[12]) are commonly used for developing druggable protein predictors (Table 2(Tab. 2)). In this section, we assessed and analyzed the performance of all available druggable protein predictors based on each benchmark dataset. 

### Performance evaluation on the Jamali2016 dataset

Jamali et al. (Jamali et al., 2016[17]) created the Jamali2016 dataset containing 1,224 positives and 1,319 negatives (Table 2(Tab. 2)). Six state-of-the-art druggable protein predictors, including DrugMiner (Jamali et al., 2016[17]), GA-Bagging-SVM (Lin et al., 2019[22]), DrugHybrid_BS (Gong et al., 2021[13]), XGB-DrugPred (Sikander et al., 2022[36]), Iraji's method (Iraji et al., 2022[16]), and DrugFinder (Zhang et al., 2023[48]), were built and evaluated based on this benchmark dataset using the 5-fold and 10-fold cross-validation tests. The performance comparison results of the Jamali2016 dataset are summarized in Table 3(Tab. 3). The prediction performance of these six druggable protein predictors was directly obtained from two literatures (i.e., Iraji et al. (2022[16]) and Zhang et al. (2023[48])). The highest ACC of 0.983 was achieved by Iraji's method, while DrugHybrid_BS and DrugFinder performed well with the second and third highest ACC of 0.966 and 0.950, respectively. In addition, Sn and Sp of Iraji's method were higher than the compared methods. 

These results indicate that Iraji's method achieved superior predictive performance in terms of the Jamali2016 dataset.

### Performance evaluation on the Yu2022 dataset

Yu et al. (Sun et al., 2018[38]) constructed the Yu2022 dataset by treating the Jamali2016 dataset as the training dataset and employing the DrugBank 5.0 database (Wishart et al., 2018[45]) and Kim's study (Kim et al., 2017[18]) to construct the independent test dataset. The final training dataset of this benchmark dataset consisted of 1,224 positives and 1,319 negatives, while its independent test dataset consisted of 224 positives and 237 negatives (Table 2(Tab. 2)). Only two druggable protein predictors, including Yu's method (Yu et al., 2022[47]) and SPIDER (Charoenkwan et al., 2022[10]), were developed and assessed based on this benchmark dataset in terms of cross-validation and independent tests. The prediction performance of these two druggable protein predictors were directly obtained from the literature (Charoenkwan et al., 2022[10]). As can be seen in Table 4(Tab. 4), cross-validation results reveal that SPIDER achieved the highest ACC, Sn, MCC, and F-score of 0.919, 0.895, 0.839, and 0.914, respectively. In terms of the independent test results, SPIDER still demonstrated better performance across almost all performance metrics (i.e., ACC, Sn, MCC, and F-score). Thus, the cross-validation and independent test results on the Yu2022 dataset are sufficient to indicate that SPIDER is an accurate and stable druggable protein predictor. 

### Performance evaluation on the Chen2022 datasets

Chen et al. (2023[12]) constructed the Chen2022 dataset from the DrugBank 5.0 database (Wishart et al., 2018[45]) and the Therapeutic Target Database (TTD) (Wang et al., 2020[42]). In this benchmark dataset, Chen et al. created multiple datasets based on the E-value. Among the several datasets in the study of Chen et al. (2023[12]), two datasets, namely All-Pfam and App-Pfam, were used to develop and assess three druggable protein predictors, which include GA-Bagging-SVM (Lin et al., 2019[22]), Yu's method (Yu, et al., 2022[47]), and QuoteTarget (Chen et al., 2023[12]). The prediction performance of these three druggable protein predictors were directly obtained from the literature (Chen et al., 2023[12]). The performance comparison results are recorded in Table 5(Tab. 5). It can be observed that QuoteTarget outperformed GA-Bagging-SVM and Yu's method in terms of ACC, Sn, Sp, MCC, and F1 on both the All-Pfam and App-Pfam datasets. Specifically, QuoteTarget achieved the highest MCC of 0.900 and 0.840 on the All-Pfam and App-Pfam datasets, respectively. Meanwhile, the MCC of GA-Bagging-SVM and Yu's method on the All-Pfam and App-Pfam datasets were 0.410, 0.250 and 0.500, 0.650, respectively.

### Mechanistic interpretation of the models

The analysis of important features is able to provide a better understanding of druggable protein identification. Among the existing studies, DrugHybrid_BS (Gong et al., 2021[13]), Iraji's method (Iraji et al., 2022[16]), Yu's method (Yu et al., 2022[47]), SPIDER (Charoenkwan et al., 2022[10]), and XGB-DrugPred (Sikander et al., 2022[36]) have made efforts to determine the optimal feature sets and understand the models' output. For example, in the study of SPIDER, the genetic algorithm (GA) in conjunction with self-assessment-report (SAR) (Charoenkwan et al., 2019[11]) was used to filter informative features to construct the optimal feature set. Specifically, the Shapley Additive exPlanations (SHAP) method (Li et al., 2021[20]; Lundberg and Lee, 2017[26]; Wei et al., 2021[43]) was selected to perform the feature optimization. In particular, SHAP positive and negative values are referred to as predictions for druggable and non-druggable proteins, respectively. Charoenkwan et al. (2022[10]) mentioned that LR-RSsecond, LR-DPC, SVM-AAC, SVM-RSpolar, and PLS-RScharge were listed as the top five important features in terms of SHAP value. Their analysis results reported that LR-RSsecond, LR-DPC, SVM-AAC, and SVM-RSpolar had positive SHAP values indicating that they contribute to the prediction of druggable proteins. As a result, for a new unknown sample, if the value of LR-RSsecond of this sample is very low, then this sample will likely be classified as a non-druggable protein; otherwise, it will be classified as a druggable protein.

### Webserver and code availability

To date, numerous studies have mentioned that developing webservers play an important role in facilitating experimental researchers to carry out their experimental analyses (Charoenkwan et al., 2022[4], 2023[5][8]; Li et al., 2021[20]). However, only two existing computational approaches (i.e., DrugMiner (Jamali et al., 2016[17]) and SPIDER (Charoenkwan, et al., 2022[10])) were deployed as webserver, while five existing studies (i.e., GA-Bagging-SVM (Lin et al., 2019[22]), XGB-DrugPred (Sikander et al., 2022[36]), Yu's method (Yu et al., 2022[47]), QuoteTarget (Chen et al., 2023[12]), and DrugFinder (Zhang et al., 2023[48])) provided their source codes (Table 6(Tab. 6); References in Table 6: Charoenkwan et al., 2022[10]; Chen et al., 2023[12]; Jamali et al., 2016[17]; Lin et al., 2019[22]; Sikander et al., 2022[36]; Yu et al., 2022[47]; Zhang et al., 2023[48]). Please note that, among the five existing studies, the source code of XGB-DrugPred is not accessible (at https://github.com/wangphd0/drug). In contrast, the DrugMiner source code is publicly available at http://www.drugminer.org/. DrugMiner was developed using NN in conjunction with top-130 informative features, but its evaluation was based solely on the cross-validation test, limiting its applicability for practical use. On the other hand, SPIDER was evaluated using both the cross-validation and independent tests, and its source code is publicly available at http://pmlabstack.pythonanywhere.com/SPIDER. The cross-validation and independent test ACC for SPIDER were 0.919 and 0.907, respectively (Table 4(Tab. 4)). Overall, it can be concluded that SPIDER outperformed that other existing approaches in terms of predictive accuracy.

## Current Limitations and Future Improvements

In this section, we aim to discuss the current limitations of the ten existing state-of-the-art predictors and provide useful guidanceto the scientific community in the design and development of more accurate, robust, and stable prediction models for in silico prediction of druggable proteins. First, data redundancy is one of the most important factors for model development (Charoenkwan et al., 2021[3]; Wei et al., 2018[44]). The current training datasets used to develop the existing methods contained redundant samples. Thus, it could be inferred that the existing methods might not provide stable and robust performance in some cases. To improve the stability and robustness of the models, it is desirable to construct a high-quality dataset by removing redundant samples using the CD-HIT tool (Li and Godzik, 2006[21]). Second, the interpretability of the existing methods remains unsatisfactory. As mentioned above, few existing methods, including Yu's method (Yu et al., 2022[47]) and SPIDER (Charoenkwan et al., 2022[10]), achieved impressive performance in both the cross-validation and independent tests. However, these methods cannot directly provide a better understanding of druggable proteins (Liou et al., 2015[24]; Vasylenko et al., 2015[40]). Recently, Charoenkwan et al. (2023[5][8]) introduced a novel propensity score representation learning (PSR) method for the identification and analysis of several proteins and peptides. In the PSR method, it is capable of generating the propensities of amino acids and dipeptides in a supervised manner. Additionally, PSR-derived propensity scores are able to elucidate the relationship between proteins/peptides and their essential physicochemical properties. In the future, we are motivated to employ the PSR method for developing an interpretable druggable protein predictor. Last, a webserver that can predict druggable proteins based on sequence information will greatly facilitate large-scale identification. To date, numerous attempts have been made to develop more accurate and stable druggable protein predictors. However, they have not been deployed as webservers or stand-alone software, limiting their utilization. It is recommended that more online webservers are highly needed to be developed to serve the community-wide efforts in identifying new druggable proteins.

## Conclusions

In this study, we provide the first comprehensive survey regarding the state-of-the-art computational approaches for *in silico* prediction of druggable proteins. Specifically, we discussed the advantages and disadvantages of the state-of-the-art computational approaches, considering a variety of important aspects that are beneficial for developing an efficient and stable prediction model. These aspects include benchmark datasets along with feature extraction schemes, ML strategies, evaluation methods, and webserver availability. Among the state-of-the-art computational approaches, the experimental results demonstrated that SPIDER was able to provide a more reliable performance in terms of both the cross-validation and independent test results. In addition, this approach has been deployed as a user-friendly webserver, accessible at http://pmlabstack.pythonanywhere.com/SPIDER. Although QuoteTarget, Yu's method, and Iraji's method can produce great performance, their utilization for large-scale identification is limited. Based on our comparative analysis, it can be demonstrated that the SPIDER approach is deemed as the best computational approaches in terms of prediction performance and usability.

## Declaration

### Ethical statement

This review paper does not include animal or human experiments.

### Conflicts of interest

The authors declare no conflict of interest.

### Author contribution's statement

WS: Project administration, supervision, designing the study, formal analysis, visualization, investigation, preparation of the manuscript, revision of the manuscript. NS: Revision of the manuscript. JN: Preparation of the manuscript. All authors reviewed and approved the manuscript.

### Acknowledgments

This work was fully supported by Mahidol University and Faculty of Medical Technology, Mahidol University.

### Funding

This project is funded by the National Research Council of Thailand and Mahidol University (N42A660380), and Specific League Funds from Mahidol University.

## Figures and Tables

**Table 1:**
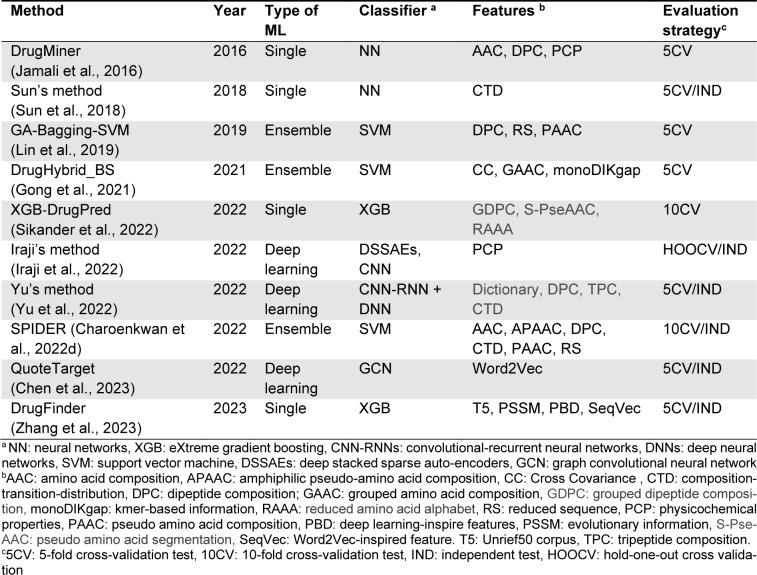
Summary of existing methods and tools for prediction of druggable proteins

**Table 2:**
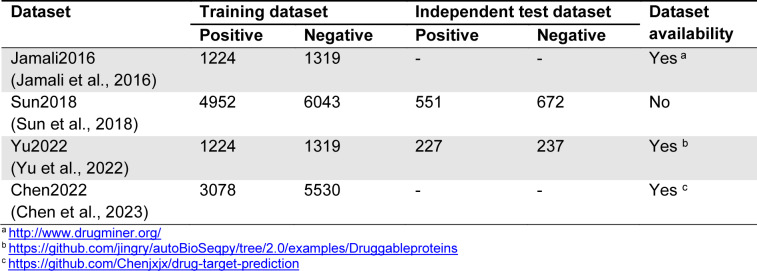
A summary of three benchmark datasets used in the existing methods

**Table 3:**
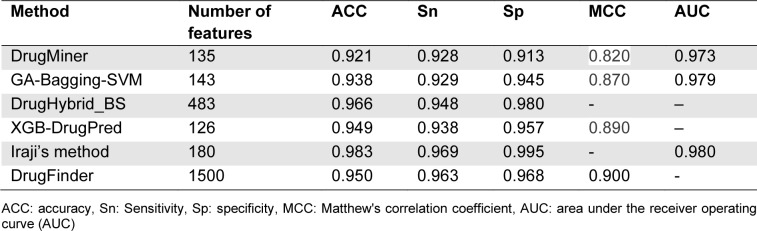
Performance comparison of DrugMiner, GA-Bagging-SVM, DrugHybrid_BS, XGB-DrugPred, Iraji's method, and DrugFinder on the Jamali2016 dataset

**Table 4:**
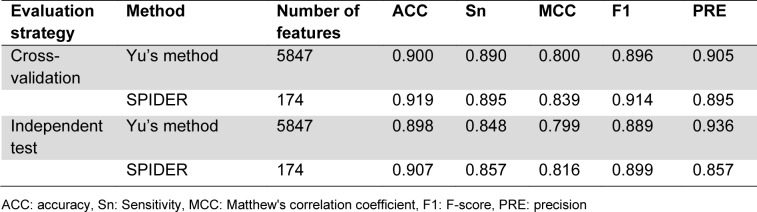
Performance comparison of Yu's method and SPIDER on the Yu2022 dataset

**Table 5:**
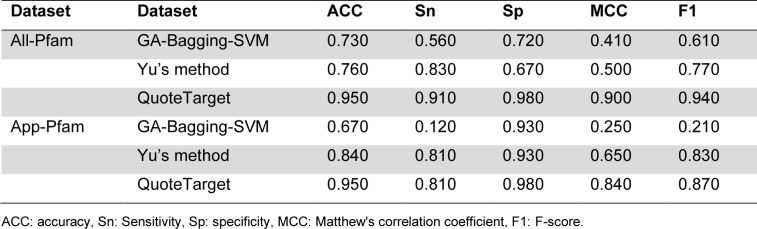
Performance comparison of Yu's method and SPIDER on the Yu2022 dataset

**Table 6:**
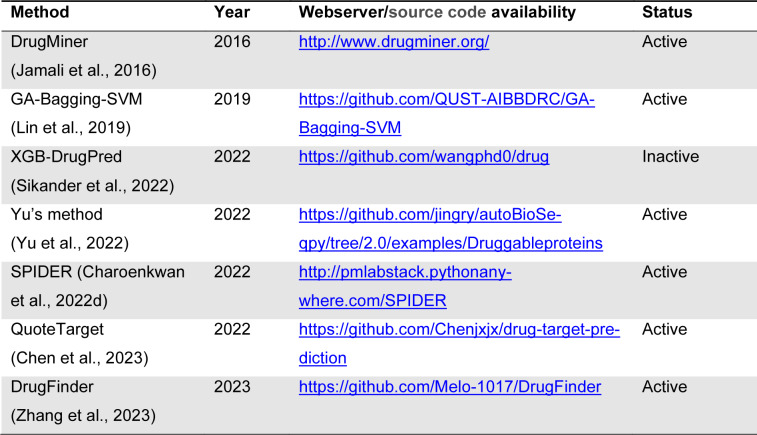
Summary of web server/source code availability for druggable protein identification
